# Effect of Water Avoidance Stress on serum and urinary NGF levels in rats: diagnostic and therapeutic implications for BPS/IC patients

**DOI:** 10.1038/s41598-019-50576-4

**Published:** 2019-10-01

**Authors:** Bruno Dias, Paula Serrão, Francisco Cruz, Ana Charrua

**Affiliations:** 1 Departamento de Biomedicina -Unidade de Biologia Experimental, Faculdade de Medicina da Universidade do Porto, Porto, Portugal; 2 Departamento de Urologia, Centro Hospitalar de São João, Porto, Portugal; 3 Departamento de Biomedicina - Unidade de Farmacêutica e Terapêutica, Faculdade de Medicina da Universidade do Porto, Porto, Portugal; 4 MedInUP – Center for Drug Discovery and Innovative Medicines, University Porto, Porto, Portugal; 5 Serviço de Urologia Faculdade de Medicina da Universidade do Porto, Porto, Portugal; 6 i3S - Instituto de Investigação e Inovação em Saúde, Universidade do Porto, Porto, Portugal; 7 IBMC – Instituto de Biologia Molecular e Celular, Universidade do Porto, Porto, Portugal

**Keywords:** Urology, Neuroscience

## Abstract

Nerve growth factor (NGF) is thought to play a key role in chronic pain felt by bladder pain syndrome/interstitial cystitis (BPS/IC) patients by activating its high affinity receptor tropomyosin-related kinase subtype A (Trk A). Whether this pathway is also involved in the aggravation of pain sensation during stress events was here investigated. The levels of plasmatic NGF were increased in rats submitted to Water Avoidance Stress test (WAS), compared to controls. The administration of the alpha1A adrenoceptors blocker silodosin prevented the increase of plasmatic NGF. Urinary NGF levels were also moderately increased in animals submitted to WAS. WAS increased pain behaviour score, lowered abdominal mechanical pain threshold and increase voiding bladder reflex activity. These changes were prevented by the administration of TrkA antagonist GW441756. These findings prompt the use of plasmatic NGF as diagnosis tool for chronic visceral painful conditions and opens therapeutic opportunities for TrkA receptors antagonist/NGF sequestration.

## Introduction

NGF is a master modulator of neural plasticity in particular of nociceptive peptidergic neurons, by recruiting, phosphorylating and activating the tropomyosin-related kinase subtype A (TrkA) receptor^1^. Multiple studies have described that the urine of IC/BPS patients and of rodent models of this human disease have elevated levels of nerve growth factor (NGF) incompared to controls^2–6^. NGF is also elevated in the serum of these patients, although this fact has been infrequently reported^7^. In a recent pooled analysis from 3 small clinical trials, women with BPS/IC and with symptoms suggesting the concomitant presence of non-urological somatic painful syndromes are those that benefit more from NGF sequestration treatment^8^. Therefore, it is not surprising that the sequestration of NGF by its highly specific humanized monoclonal antibody is under investigation to manage bladder pain in BPS/IC patients.

The high levels of NGF observed in BPS/IC patients have been attributed to the excess of synthesis and release of the neurotrophin from the bladder urothelium and detrusor smooth muscle cells^9,10^. In this hypothesis, the high urinary levels of NGF results from its diffusion from the bladder wall into the urine and the NGF serum levels would be the consequence of its diffusion from the bladder tissue into the blood vessels.

Typically, BPS/IC patients report an increase in bladder pain intensity during stressful events^11^. If such events cause an increase of NGF in the urine or in the serum, therefore contributing to pain exacerbation, it is presently unknown. Nevertheless, this information can be highly relevant in order to determine if patients under stressful conditions also benefit from NGF sequestering strategy. It may be recalled that BPS/IC patients also show an increase of noradrenaline (NA) levels in the plasma and in the urine^12^. Excess of noradrenaline may lead by itself to pain enhancement by activating the numerous alpha-adrenoceptors expressed by nociceptive sensory fibers^13^.

To clarify the role of NGF in pain exacerbation during stressful conditions, in the present work we used an animal model of chronic stress, the Water Avoidance Stress (WAS), a rat model that is known to course with intense, prolonged bladder pain. We investigated the serum and urinary levels of NGF and the effect of the blockade of TrkA, the high affinity NGF receptor, in bladder pain and bladder function.

## Results

### NGF levels determination by ELISA

**Figure 1:**
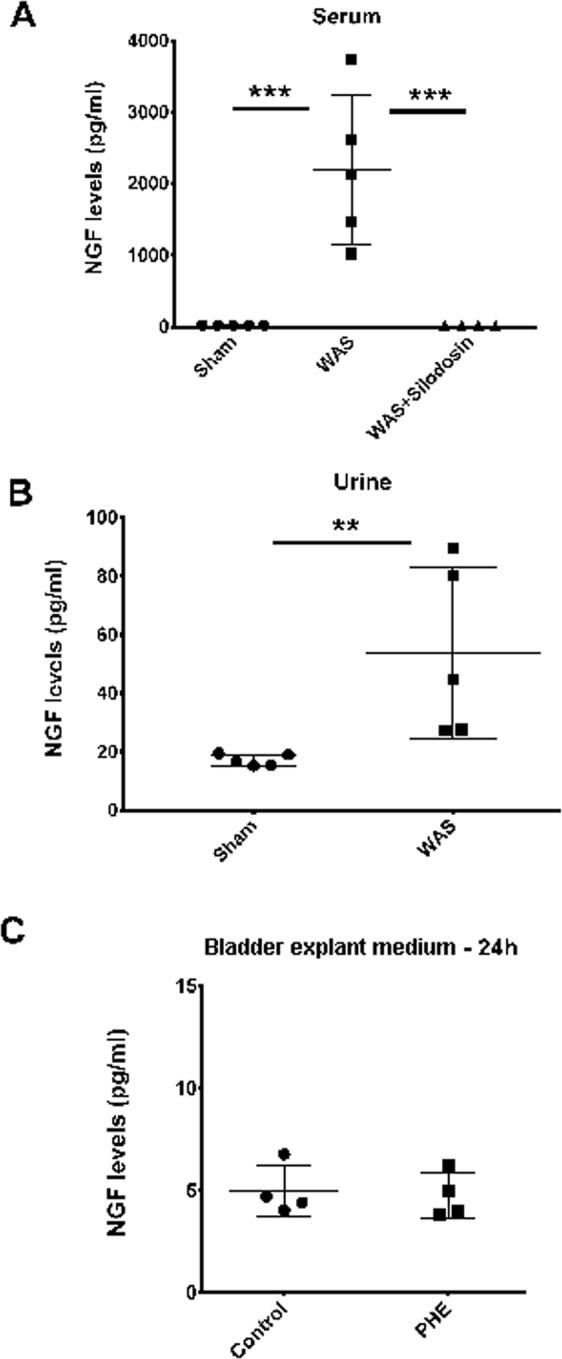
Scatter graph showing the NGF levels (pg/ml) in **(A)** serum of sham, WAS and WAS + Silodosin groups; **(B)** urine of sham and WAS groups; **(C)** bladder explant medium after 24 h of exposition to medium (control) and phenylephrine (PHE). **p < 0.01; ***p < 0.001.

Plasmatic levels of NGF on sham group were 19.18 ± 0.47 pg/ml (Fig. 1A). WAS induced a marked increase in plasmatic NGF levels to 2194.17 ± 942.73 pg/ml (Fig. 1A; ANOVA followed by Tukey’s multiple comparison test: p < 0.0001). Concerning urinary NGF, animals from sham group presented 17.17 ± 1.73 pg/ml (Fig. 1B). In the WAS group, there was a moderate increase of the urinary NGF to 53.91 ± 26.26 pg/ml (Fig. 1B; unpaired T-test: p = 0.0235).

The administration of the apha1A antagonist silodosin during WAS induction prevented the increase in serum NGF levels (17.50 ± 0.53 pg/ml; ANOVA followed by Tukey’s multiple comparison test: p = 0.0001 compared to WAS and p = 1 compared with sham).

In the explant experiments, the culture medium of the non-stimulated bladder strips (control) contained 4.97 ± 1.06 pg/ml.mg bladder strip (Fig. 1C). The stimulation with 1.25 g/l of phenylephrine did not induce any change in the NGF released to the media (4.75 ± 0.94 pg/ml.mg bladder strip; Fig. 3C; Mann-Whitney Test: p = 0.6571).

### Evaluation of pain behaviour

**Figure 2:**
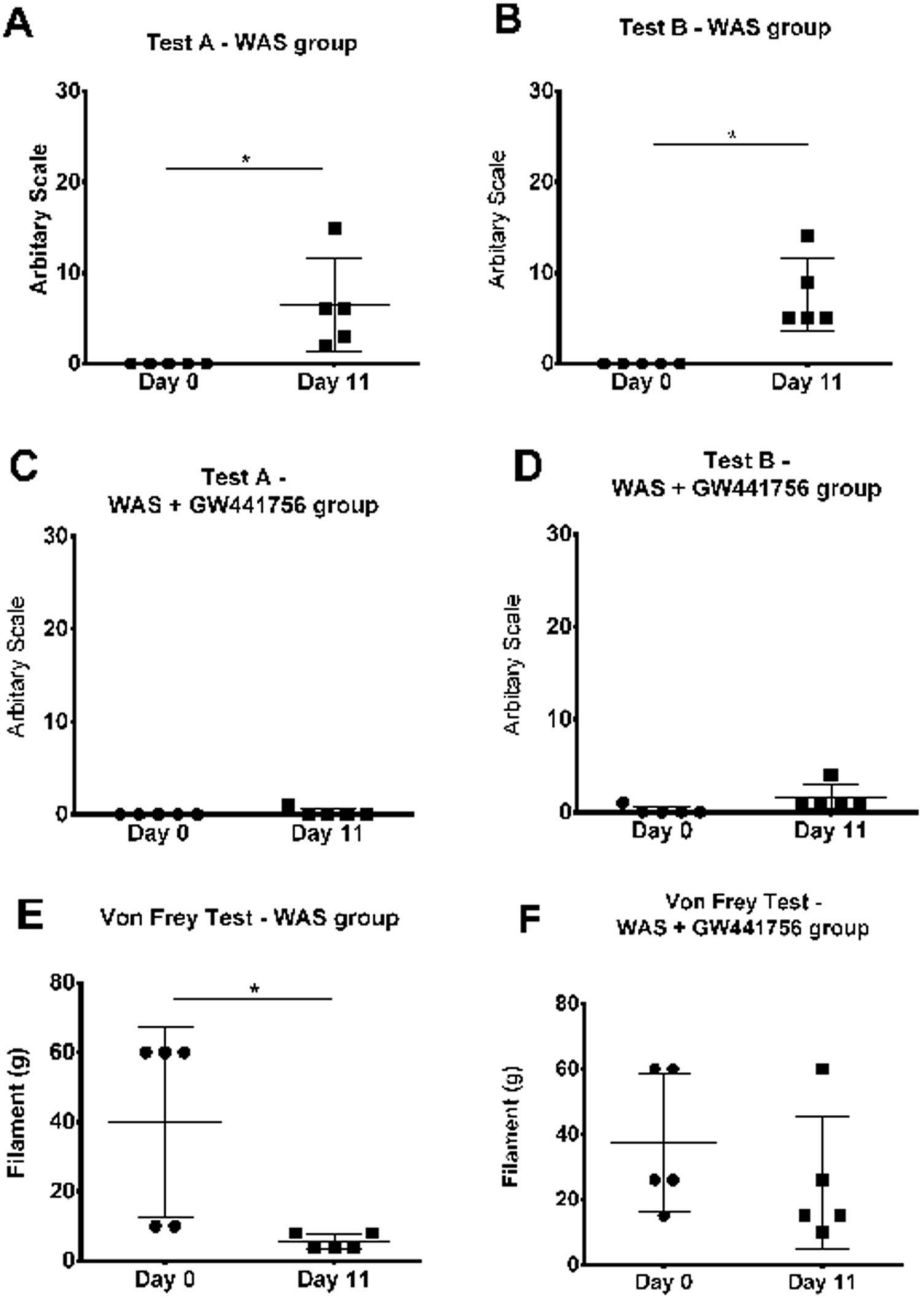
Scatter graph showing pain related behaviour arbitrary values before (day 0) and after (day 11) WAS induction for test A **(A**) and test B **(B)**. Scatter graph showing pain related behaviour arbitrary values before (day 0) and after (day 11) WAS+ Trk A antagonist GW441756 (WAS+ GW441756) administration for test A **(C)** and test B. (**D**) Scatter graph showing the lower abdominal mechanical pain threshold using Von Frey filaments before (day 0) and after (day 11) WAS induction **(E)** and before (day 0) and after (day 11) WAS+ Trk A antagonist GW441756 (WAS + GW441756) administration **(F)**. *p < 0.05.

The WAS group increase the pain score from day 0 to day 10, from values of 0.0 ± 0.0 to 6.4 ± 4.6 in test A (Fig. 2A; paired t test: p = 0.0493), and from values of 0.0 ± 0.0 to 7.6 ± 3.6 in test B (Fig. 2B; paired t test: test B, p = 0.0129). In the WAS animals treated daily with GW441756, a specific TrkA antagonist, the pain score was maintained in baseline values throughout the experiment for both tests. For test A, the values were 0.0 ± 0.0 at day 0 and 0.2 ± 0.4 at day 10 (Fig. 2C; paired t test: p = 0.3739). For test B, the values were 0.2 ± 0.4 at day 0 and 1.6 ± 1.2 at day 10 (Fig. 2D; paired t test: p = 0.1079).

The mechanical pain threshold of WAS group decrease from day 0 basal value of 40 ± 24 g to day 10 value of 6 ± 2 g (Fig. 2E; paired t test: p = 0.0415). The daily administration of GW441756 prevented the decrease in mechanical pain threshold. At day 0 the animals presented abdominal mechanical pain threshold of 37 ± 19 g and at day 10 the mechanical pain threshold was 25 ± 18 g (Fig. 2F; paired t test: p = 0.2374).

### Bladder reflex activity evaluation by cystometry

**Figure 3:**
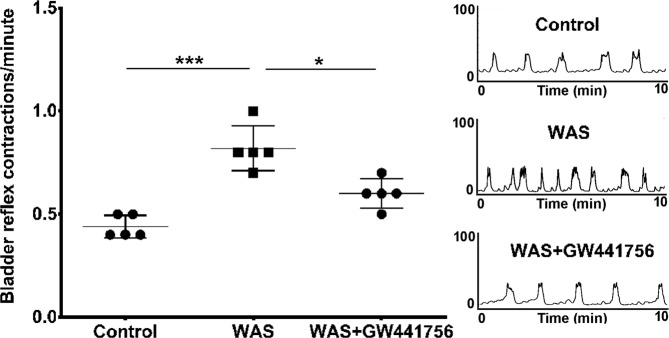
Scatter graph showing the variation of voiding frequency of control animals, animals submitted to WAS and animals submitted to WAS treated with Trk A antagonist GW441756 (WAS+ GW441756) (*p < 0.05; ***p < 0.001). Typical cystometrogram are presented for each group of animals (Y axis – Pressure (cm H_2_O), X axis – Time (minutes).

Sham animals had 0.44 ± 0.05 bladder voiding reflex contractions/minute. The animals from WAS group presented 0.82 ± 0.10 bladder voiding reflex contractions/minute (Fig. 3; ANOVA followed by Tukey’s multiple comparison test: p = 0.0008). The administration of GW441756 decreased bladder reflex contractions to 0.60 ± 0.0 (Fig. 3; ANOVA followed by Tukey’s multiple comparison test: p = 0.0186 compared with WAS group and p < 0.05 compared with sham group).

### Noradrenaline levels determined by HPLC

**Figure 4:**
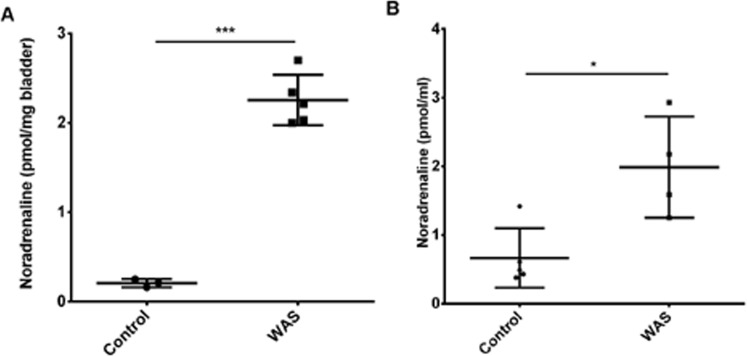
Scatter graph showing the noradrenaline levels in **(A)** the bladder (pmol/mg) and **(B)** serum (pmol/ml) of animals from control and WAS groups. *p < 0.05; ***p < 0.001.

The bladder of sham group had 0.21 ± 0.03 pmol of noradrenaline/mg of bladder homogenate (Fig. 4A). After WAS, animals presented 2.26 ± 0.26 pmol of noradrenaline/mg of bladder (p < 0.001; Fig. 4A). Plasmatic levels of noradrenaline in the sham and WAS groups were 0.66 ± 0.35 nmol/ml and 1.99 ± 0.64 nmol/ml, respectively (p < 0.05; Fig. 4B).

## Discussion

The most important finding of our study was the marked increase of NGF in the serum after the exposure of naïve female rats to WAS condition. In animals stimulated by WAS test, serum NGF levels increased 114 times, by far exceeding the 3 times increment observed in the urine of the same animals. Among the consequences of the systemic increase of the neurotrophin, a strong sensitization of the nociceptors throughout the body of the animals may have occurred, explaining the intense pain behaviour, the decrease of mechanical pain threshold in lower abdominal and the duplication of the number of bladder voiding reflex contractions. All this changes were dependent upon the activation of the TrkA receptors, the high-affinity receptors for NGF and consequent nociceptors sensitization. In fact, the blockade of TrkA with its specific antagonist GW441756 prevented the manifestations seen in non-treated WAS animals.

The levels of NGF in the urine of WAS animals was much more modest than in serum. These experimental findings agree with observations in BPS/IC patients in whom the mean NGF serum levels were 3.48 pg/mL while the mean urinary levels, corrected for creatinine, were only 0.69 pg/mg^14^. These observations raise the hypothesis that the source of the neurotrophin may be other than the bladder wall. The marked increase of serum NGF may in fact reflect a systemic increase of the neurotrophin rather than a bladder synthesis and its posterior release into the serum and urine. It would be difficult to support that the bladder, able to increase the urinary levels by 3 times, were able to increase the serum levels by 114 times. The appearance of NGF in the urine could, in this hypothesis, result of its diffusion from bladder blood vessels into the urine. In addition, due to its low molecular weight (13.2 kDa, five times less than albumin molecular weight), NGF can appear in urine after filtration in renal glomeruli. Moreover, the fact that 24 h stimulation with phenylephrine intended to mimic the stressful conditions in culture experiments did not change NGF levels in culture medium containing our whole bladder strips reinforces the hypothesis that the serum NGF detected in the blood of WAS rats was synthetized outside the bladder. It may be recalled that the time of exposure to phenylephrine in culture experiments were shown to be sufficient for catecholamines to induce a robust release of NGF from non-neuronal cells into culture medium^15^.

If the above experimental and clinical observations may not support the bladder tissue as the main source for the increased serum levels of NGF, it might be important to discuss other possible origins for the neurotrophin. Mast cells have been pointed out as another important source of NGF^16^. Mast cells have been shown to be increased in the bladder tissue of BPS/IC patients which course with a heavy inflammation of the organ, but not in other organs of BPS/IC patients. Therefore, mast cells are an unlikely source for the systemic increase of NGF in WAS rats. Other cells that may synthetize and release NGF are fibroblasts. These cells, in *in vitro* experiments, were shown to release NGF into the culture media though a process that, interestingly, was stimulated by catecholamines^15^. These findings agree well with our present findings. The rat model of WAS courses with increased levels of noradrenaline in the serum and in the bladder wall.

The administration of silodosin, an alpha 1A adrenoceptor antagonist was shown to prevent NGF increase, to supress pain behaviour, to normalize mechanical pain threshold and bladder reflex voiding contractions in WAS rats. It may be recalled that in previous studies, we showed that chronic exogenous administration of an adrenergic agent, phenylephrine, to intact rats induced a robust spinal cord expression of c-fos, a surrogate marker of pain, after innocuous bladder stimulation. Spinal c-fos expression was prevented by the administration of alpha1A adrenoceptor antagonists^13^.

Among the most frequently used models of chronic bladder pain in rodents are the ones in which the urothelium is severely damaged with cyclophosphamide or protamine. In these models an intense pan-mural inflammation swiftly develops in the bladder. In these models a rapid increase of NGF and other inflammatory mediators is expected to occur^17^. Unfortunately, in these models the serum NGF was not systematically measured for comparison with the urinary levels. However, taking in consideration the nature of the models it is expectable that the results might be substantially different from those obtained in the WAS model used in the present study. Comparisons among rodent models of BPS/IC are lacking but are utterly necessary in order to compare the outcomes reported.

The implications of our observations are multiple. Serum NGF may become an important biomarker to phenotype BPS/IC patients. Being a disease which has no defined etiology yet, grouping patients with similar characteristics may foster the process of discovery. In fact, the etiopathogenesis is better recognized in homogeneous than in heterogeneous groups. NGF measurement in the blood may help to identify patients that respond better to humanized antibodies presently under development to sequester an excess of NGF. Most likely, patients with high levels of the neurotrophin will be the best responders. Recent studies showed that women with BPS/IC and associated non-urological somatic syndromes were more likely to experience significant pain reduction with antibodies anti-NGF^2,18^. Those characteristics may well be a consequence of high NGF levels in the blood leading to a systemic sensitization of somatic and non-bladder visceral nociceptors. It is important to mention that in a small cohort of 28 BPS/IC patients, an elevated serum NGF was detected in only 17 (60%) of them, potentially reinforcing the importance of NGF as a biomarker for patient phenotyping^19^. The present study also suggests that TrkA antagonist may be used in lieu of anti-NGF antibodies. In addition to the obvious facility in its administration (NGF antibodies require sub-cutaneous injections while TrkA antagonists are expected to work easily by oral route) TrkA antagonists may also be useful by intravesical route, as long as small molecules that cross the urothelium can be produced.

The capacity of silodosin to prevent the increase in plasmatic NGF and at the same time to avoid the development of pain behaviour, bladder reflex hyperactivity and decrease in abdominal mechanical pain threshold in animals exposed to WAS may suggest that this alpha blocker can be useful in the pain management of some BPS/IC women, in particular those showing evident signs of stress and non-urological symptoms. This drug has been extensively investigated in men with lower urinary symptoms associated with enlarged prostates and did not show any serious adverse events during its administration^20^. Therefore, a trial with silodosin in women with BPS and signs of stress may be of interest.

The activation of TrkA receptors influences the expression of other pain receptors^21^. A previous study showed that NGF-induced bladder overactivity and bladder noxious input was depending on the interaction of NGF with TRPV1^22^. In fact, NGF had no effects of TRPV1 KO mice^22^. TRPV1 synthesis and transport to the periphery is stimulated by NGF^23^. If TRPV1 represents a bottleneck pathway in bladder pathologies associated with NGF up-regulation, TRPV1 antagonists may demonstrate therapeutic relevance in BPS/IC patients as well. In fact, earliest TRPV1 antagonists available were shown to reduce bladder pain and bladder reflex activity in rodent models of chemical bladder inflammation^24^.

In conclusion, our exploratory study demonstrates that chronic stress causes an increase of NGF in the serum and in the urine. Being the levels in the former much higher than those in the urine, it is unlikely that the blood NGF has its origin only in the bladder. Identification of extra-bladder tissues able to synthetize and release NGF in patients with BPS/IC is therefore necessary. Finally, NGF sequestration or TrkA antagonists is supported as an important therapy for BPS/IC.

## Methods

This work was approved by the animal ethical committee “ORBEA - Organismo Responsável pelo Bem-Estar dos Animais” from our institution as well as by the national animal ethical committee DGAV. All experiments were performed in accordance with relevant guidelines and regulations.

### Animal model

A group of five adult female Wistar rats (200–225 g) obtained from our animal facility were submitted to Water Avoidance Stress (WAS) test (WAS group), as recently described. Briefly, animals were placed on pedestals (cylinder 52π × 14 cm^3^) in the centre of cages (26.6 × 42.5 × 18.0 cm^3^), filled with water at room temperature, for 1 h/day, for ten consecutive days. The experiments were performed between 10:00 a.m. and 12:00 a.m. to minimize circadian effects. The experiments were performed under red light as animals were housed in inverted light cycle.

Another group of five adult female Wistar rats were submitted to WAS while orally received the TrkA antagonist GW441756, 58 µg/kg/day (Biotechne).

Another group of four adult female Wistar rats were submitted to WAS while orally received the alpha 1 A adrenoceptor silosodin at 0.2 mg/kg.day (commercial formulation available from Recordati Urorec®). This group was only used to collect blood for the determination of NGF levels.

Another group of five adult female Wistar rats were placed on the pedestals in waterless cages (Sham group). This group was only used as control group for cystometries and for NGF determination in serum and urine.

Another group of four adult female Wistar rats was used only for explant cultures experiments (intact group).

### Bladder explant culture experiments

The bladders of intact rats were collected, and bladder strips were cut and left in a urothelial cell culture medium (Cat No 4321; ScienCell), in culture medium with 1.25 g/l of phenylephrine (P6126, Sigma) or in culture medium with 1.25 g/l of phenylephrine and 0.1 g/l of the alpha 1 A adrenoceptor blocker silodosin (commercial formulation available from Recordati Urorec®). After 24 h, the medium was collected to measure NGF by ELISA.

### NGF levels evaluation by ELISA

Plasma, urine and culture media were used to analyse NGF levels by ELISA, following the manufactures instruction (ELH-BNGF RayBio Human beta-NGF Kit; minimum sensitivity is 14 pg/ml). The manufacture informed that this ELISA kit shows no cross-reactivity with the following cytokines tested: human Angiogenin, BDNF, BLC, ENA-78, FGF- 4, IL-1 alpha, IL-1 beta, IL-2, IL-3, IL-4, IL-5, IL-6, IL-7, IL-8, IL-9, IL-10, IL-11, IL-12 p70, IL-12 p40, IL-13, IL-15, I-309, IP-10, G-CSF, GM-CSF, IFN-gamma, Leptin (OB), MCP-1, MCP-3, MDC, MIP-1 alpha, MIP-1 beta, MIP-1 delta, MMP-1, - 2, -3, -10, PARC, RANTES, SCF, TARC, TGF-beta, TIMP-1, TIMP-2, TNF-alpha, TNF-beta, TPO, VEGF. No other proteins were tested in the present work.

Briefly, soluble NGF from samples bind to anti-NGF coated plates. Captured NGF was recognized by a biotinylated secondary antibody that was combined with HRP conjugated streptavidin. The chromogenic substrate 3,3,5,5′-tetramethylbenzidine was added for colour development and sulphuric acid was used to stop the reaction. The amount of NGF in the sample was measured at 450 nm with a Synergy HT Microplate Reader (BioTek Instruments, USA). All samples and standards were run in duplicate and values were averaged against a standard curve generated with known amounts of NGF.

### Noradrenaline evaluation

Bladder and urine were used to analyse noradrenaline levels by HPLC. Urines were acidified with HCl 6 M and immediately stored at −20 °C, until urine of all groups was gathered and then transferred to −80 °C. Noradrenaline levels were determined by HPLC with electrochemical detection, as previously described^25^. Briefly, aliquots of urine were added to 50 mg alumina (to adsorb noradrenaline) and the pH was adjusted to pH 8.3–8.6 with TRIS buffer. Then noradrenaline was eluted with 200 μl of 0.2-M perchloric acid on Costar Spin-X microfilters, and 50 μl of the eluate was injected into an HPLC (Gilson Medical Electronics, Villiers le Bel,France). The lower limit of detection of noradrenaline was 350 fmol.

### Evaluation of pain behaviour

After an initial period of habituation (repeated 5 days before day 0) to the experimental environment and to handler, visceral pain behavioural tests and lower abdomen pain were evaluated on WAS and WAS + GW441756 groups, using a mechanical hyperalgesia test previously described. Briefly, to evaluate visceral pain behaviour, two test were performed. In test A, animal breathing rate, eyes aperture and body posture using were analysed at day 0 (control) and at day 10. An arbitrary 0–10 scale was used for each parameter. For the breathing rate, every decrease of 10 cycle/minute compared with day 1 record was scored 1. For the eye aperture test, the following scale was used: 0 for complete opening, 2 for intermediate opening, 5 for half-closed, 7 for intermediate opening and 10 for complete closing. For body posture, if animals presented rounded-back with body aligned or motionlessness the score was 10. Otherwise the score was 0. In test B, the following parameters were scored: normal 0, piloerection 1, strong piloerection 2, laboured breathing 3, licking the abdomen 4 and stretching and contractions of the abdomen 5.

To evaluate mechanical hyperalgesia, at day 0 and day 10, animals were placed in individual chambers with a wire mesh floor and allowed to acclimate. Then, a series of Von Frey monofilaments (rated from 4 to 60 g) were applied perpendicularly in the lower abdomen, with enough strength to cause the monofilament to slightly bend. Each monofilament was tested five times, with 5 s interval between each application. If there was abdomen retraction or the animal jump in at least three of the five filament applications, it was considered a positive response. In case of no response to the filament, the next-stronger monofilament was applied within an interval of 30 s.

### Bladder reflex activity evaluation by cystometry

At day 11, after urine collection from sham and WAS groups, animals from sham, WAS and WAS + GW441756 groups were anaesthetised with urethane (1.2 g/kg body weight) laid on a heating pad with a rectal probe inserted to measure body temperature. The bladder was then exposed through a low abdominal incision. A 20-gauge needle was inserted in the bladder dome and saline was infused (6 ml/h). After a stabilization period of 30 min, the cystometrograms were recorded for 10 min. Cystometrograms were analysed, and bladder reflex activity was expressed as the number of bladder contractions per minute. After the cystometry, blood was collected from the heart from sham, WAS and WAS + GW441756 groups, using a needle coated with heparin and centrifuged at 1000 g for 15 minutes and plasma was collected and stored at −20 °C to measure NGF by ELISA and noradrenaline by HPLC. Blood was also collected from animals of WAS + silodosin group to measure NGF by ELISA. Afterwards, animals were euthanized.

### Statistical analysis

Pain behaviour score, mechanical pain threshold, bladder reflex activity and NGF values are represented as mean ± standard deviation. Statistical analysis was performed using Graph Pad Prism 7. When comparing two groups, significance was estimated using Mann-Whitney Test. When comparing more than two groups, significance was estimated using ANOVA followed by Tukey’s multiple comparisons test, as all data showed to follow a normal distribution and to minimize Type I errors (false positive).
